# Personnel knowledge of intravenous admixtures: a survey in a government hospital

**DOI:** 10.11604/pamj.2021.40.198.30093

**Published:** 2021-12-01

**Authors:** Erza Genatrika, Ika Puspitasari, Susi Ari Kristina, Teuku Nanda Saifullah Sulaiman

**Affiliations:** 1 Faculty of Pharmacy, Universitas Gadjah Mada, Yogyakarta, Indonesia,; 2 Faculty of Pharmacy, Universitas Muhammadiyah Purwokerto, Central Java, Indonesia

**Keywords:** Knowledge, personnel, intravenous, admixtures

## Abstract

**Introduction:**

in Indonesia, intravenous admixtures are a common problem in hospitals. The incidence of microbial contamination in hospitals is still increasing every year. As such, knowledge of compounding personnel about intravenous admixtures is crucial in determining product quality. This study aims to assess the compounding personnel´s knowledge regarding intravenous admixtures and determine the relationship between socio-demographic characteristics with knowledge of compounding personnel in a government hospital, Indonesia.

**Methods:**

a cross-sectional study was conducted on 119 compounding personnel selected using purposive sampling from five different hospital units from September to November 2020. Data were collected using a self-administered questionnaire on socio-demographic factors and knowledge of intravenous admixtures.

**Results:**

of the 119 compounding personnel who was respondent in this study, only 28 compounding personnel had good knowledge (23.5%). Most of the respondents were female at 52.9%, early adulthood at 63.9%, profession as a nurse of 100%, working period less than five years at 37.0%, civil servants at 53.8%, and employees who have never attended training at 84.9%. Spearman rank correlation test results showed that no significant correlation between sex, profession, working period, and employment status with knowledge. However, age and intravenous admixtures training history have a significant correlation with knowledge.

**Conclusion:**

we found that most compounding personnel in a government hospital, Indonesia, were sufficiently understood with intravenous admixtures so that they should be aware of the importance of performing intravenous admixtures adequately.

## Introduction

Injectable drugs are the pharmaceutical preparation most frequently given to hospitalized patients around the world. Injectable drugs can be given alone or in the form of a mixture with other parenteral preparations. Intravenous (i.v) admixtures are the mixing of two or more parenteral preparations in a hospital to meet an individual patient´s therapeutic needs. The mixing process of parenteral preparations is a complex process with various risks that arise if it is not prepared aseptically. One of the risks is bacterial contamination, and contaminated parenteral preparations can cause severe patient complications [1-6]. The risk of bacterial contamination is often related to the limited knowledge of personnel regarding the procedure of (i.v) admixtures.

Knowledge is the basis for shaping a person's behavior [7]. Besides, the knowledge of compounding personnel is also critical because only personnel who have knowledge through their participation in training on (i.v) admixture can mix parenteral preparations [8]. By having good knowledge about (i.v) admixture, it is hoped that the compounding personnel will be able to apply aseptic techniques appropriately to prevent microbial contamination. Therefore, this study aims to assess the compounding personnel´s knowledge and determine the relationship between socio-demographic characteristics with knowledge of the compounding personnel.

## Methods

**Study design and setting:** this cross-sectional study was used to study the compounding personnel knowledge and the relationship between socio-demographic characteristics with knowledge of (i.v) admixtures amongst compounding personnel in a government hospital, Indonesia.

**Study location and sample size:** the study was conducted from September to November 2020 amongst 119 compounding personnel at one of the government-owned hospitals, Purwokerto, Indonesia. A purposive sampling method was carried out to obtain to appropriate sample size that complied with the inclusion criteria, including hospital employees and personnel or health professionals who were directly involved in mixing injectable drugs.

**Data collection procedure:** the respondents were briefed about the study beforehand and willingness to fill the questionnaire was considered as consent to participate in the study. The respondents were also notified that their participation in the study was voluntary, and they can withdraw from the study at any time if they do not want to be a part of the study. Ethical principles were followed throughout the study, and ethical approval was acquired from Medical Research and Ethics Committee (approval number: 420/09770/IX/2020).

**Instruments:** the questionnaire was constructed based on a review from previous research literature of (i.v) admixtures and guidelines for compounding sterile preparations [8-11]. Five professional health experts, including three pharmacists and two lecturers in pharmacy faculty were involved in validating the questionnaire´s content. And then, the validity and reliability tests were carried out on 30 respondents to ensure the questionnaire´s overall accuracy before the full-scale research. The questionnaire comprised of two sections. Section one covers socio-demographic characteristics (sex, age, profession, working period, employee status, and (i.v) admixture training). Section two comprised of 20 questions that measured compounding personnel knowledge of (i.v) admixtures. Each question answered correctly was given a score of 1, and the question answered incorrectly, or no answer was given a score of 0. Validated research instruments can be seen in Annex 1.

**Data analysis:** all data were analyzed using SPSS version 26.0 (IBM Corporation, USA). Descriptive statistics and Spearman rank correlation was used to assess the relationship between the socio-demographic variables with knowledge of compounding personnel about (i.v) admixtures.

## Results

**Socio-demographic characteristics of the respondents:** the socio-demographic characteristics of the respondents are as in Table 1. The majority of the respondent was in the age group of 26 to 35 (63.9%), profession as a nurse (100%), female (52.9%), working period 1 to 5 years (37%), civil servants (53.8%), and who have never attended training (84.9%).

**Table 1:** socio-demographics of respondents

		Frequency	Percent (%)	Cumulative percent (%)
Sex	Female	63	52.9	52.9
	Male	56	47.1	100.0
Age	17-25^(1)^	7	5.9	5.9
	26-35^(2)^	76	63.9	69.8
	36-45^(3)^	24	20.2	90.0
	46-55^(4)^	12	10.0	100.0
	56-65^(5)^	0	0.0	100.0
Profession	Nursing	119	100.0	100.0
Working period	1-5	44	37.0	37.0
	6-10	40	33.6	70.6
	11-15	15	12.6	83.2
	16-20	7	5.9	89.1
	>21	13	10.9	100.0
Employee status	Civil servants	64	53.8	53.8
	Noncivil servants	55	46.2	100.0
Intravenous admixture training	Ever	18	15.1	15.1
	Never	101	84.9	100.0

(1): adolescents; (2): early adulthood; (3): late adulthood; (4): early age erderly; (5): late old age

**Knowledge level of (i.v) admixtures:** most of the respondents have adequate to good knowledge, and it can be seen in Table 2. There were 20 questions of knowledge about (i.v) admixtures, and most of the respondents were able to answer more than 11 questions correctly (94.1%). It means most of the respondents understand the basis of knowledge about (i.v) admixture and aspects that must be considered in (i.v) admixture, including personnel, building and equipment, procedure, packaging, and labeling, storage, distribution, and quality assurance.

**Table 2:** knowledge of compounding personnel

Knowledge level	∑ correct	Frequency	Frequency (%)
Poorly	≤11 questions	7	5.9
Adequate	12 - 15 questions	84	70.6
Good	≥16 questions	28	23.5

However, in Table 3, it is known that there are 3 aspects with the lowest percentage, namely aspects of building and equipment, the correct procedures for (i.v) admixtures, packaging, and labelling. For building and equipment, only 34.5% of respondents understand the cleanroom, 54.6% of respondents understand the anteroom. Furthermore, for a procedure, only 3 of 7 items were understood by respondents, including handwashing, watch and ring, transfer techniques (more than 50% correct). And then, most of the respondents answered wrongly for beyond used date on an aspect of packaging and labelling, only 30.3% of respondents answered correctly.

**Table 3:** knowledge based on the aspects of (i.v) admixtures

Item	Questions	Correct (%)
Basic of knowledge	Definition of aseptic compounding	97.5%
	Infusion composition	68.9%
	Solvent	96.6%
Personnel	Training	70.6%
Building and equipment	Definition of cleanroom	34.5%
	Definition of anteroom	54.6%
	Air pressure	83.2%
Procedure	Washing	92.4%
	Use of watch and ring	95.8%
	Cleaning of the working area	37.8%
	Ultra-violet lamp	45.4%
	Garbage bags	22.7%
	Gowning	19.3%
	Transfer technique	89.1%
Packaging and labelling	Label	91.6%
	Beyond used date	30.3%
Storage	Temperature	94.1%
Distribution	Use of the container	98.3%
	Use of the cool box	98.3%
Quality assurance	Documentation	97.5%

**The relationship between socio-demographic characteristic and knowledge:** a Spearman rank correlation table was carried out to determine the relationship between socio-demographic characteristics and knowledge. From Table 4, showed that a significant relationship was found between age and (i.v) admixture training with knowledge of compounding personnel (p<0.05).

**Table 4:** correlation between socio-demographic characteristics and knowledge

Variable		Knowledge level			p-value/r
		Poorly	Adequate	Good	
Sex	Female	3 (2.5%)	45 (37.8%)	15 (12.6%)	0.780/0.026
	Male	4 (3.4%)	39 (32.8%)	13 (10.9%)	
Age	17-25^(1)^	0 (0.0%)	4 (3.4%)	3 (2.5%)	0.029/0.200
	26-35^(2)^	4 (3.4%)	63 (52.9%)	9 (7.6%)	
	36-45^(3)^	2 (1.6%)	13 (10.9%)	9 (7.6%)	
	46-55^(4)^	1 (0.8%)	4 (3.4%)	7 (5.9%)	
Profession	D3 nursing	6 (5.1%)	48 (40.4%)	15 (12.6%)	0.273/0.101
	D4 nursing	0 (0.0%)	1 (0.8%)	0 (0.0%)	
	S1 nursing	1 (0.8%)	35 (29.4%)	13 (10.9%)	
Working period	1-5	2 (1.6%)	33 (27.8%)	9 (7.6%)	0.109/0.109
	6-10	2 (1.6%)	34 (28.6%)	4 (3.4%)	
	11-15	1 (0.8%)	8 (6.7%)	6 (5.1%)	
	16-20	0 (0.0%)	6 (5.1%)	1 (0.8%)	
	>20	2 (1.6%)	3 (2.5%)	8 (6.8%)	
Employee status	Civil servants	7 (5.9%)	35 (29.4%)	22 (18.5%)	0.107/0.149
	Noncivil servants	0 (0.0%)	49 (41.1%)	6 (5.1%)	
Intravenous admixture training	Never	7 (5.9%)	84 (70.6%)	10 (8.4%)	0.000/0.702
	Ever	0 (0.0%)	0 (0.0%)	18 (15.1%)	

r: correlation coefficient value; (1): adolescents; (2): early adulthood; (3): late adulthood; (4): early age erderly; (5): late old age

## Discussion

This study will describe the knowledge of compounding personnel and the relationship between socio-demographic with knowledge of compounding personnel about the aspects that must be considered in (i.v) admixtures. This study follows Arikunto's theory where a person's knowledge is categorized into three, namely personnel with poorly, adequate, and good knowledge [12]. It is known that all the compounding personnel in this government hospital are nurses and most of them have good knowledge. The compounding personnel with good knowledge are personnel who have attended training about (i.v) admixtures. Notoatmodjo (2010) states that knowledge-based behaviour will be followed by good behaviour as well [7]. Therefore, it is hoped that the knowledge already possessed by compounding personnel can be maintained so as not to experience problems in the process of (i.v) admixtures.

As for the results of the study, it was found that the compounding personnel had good knowledge but had never attended the training. Several theories have explained that the longer a person's work period will increase one's experience [7,13,14]. A person's experience at work can provide professional knowledge and skills [15]. Furthermore, compounding personnel who have poorly knowledge are those who have never attended any (i.v) admixture training. That is in line with Riris´s (2014) studies, which revealed that nurses with poor knowledge were nurses who had never participated in training [16]. Compounding personnel with poor knowledge, most of them are not familiar with aspects of (i.v) admixture procedures, such as mixing intravenous drugs must be done in a clean room, the working area must be cleaned before mixing intravenous drugs, use gowning when mixing intravenous drugs, and put trash bags in the working area. Lack of knowledge of compounding personnel about (i.v) admixtures procedures can be a factor in microbial contamination [11].

In addition to their lack of knowledge on procedures, the compounding personnel also do not understand about beyond use date (Table 3). Beyond use date (BUD) and expiration date are not the same [8]. Chemical and physical stability must be considered when assigning a BUD. Therefore, it is very important to improve the knowledge of compounding personnel about BUD, so that the intravenous drugs given do not endanger patient safety [17,18]. The current study reflected a positive association between the (i.v) admixture training with knowledge of compounding personnel regarding (i.v) admixtures (r=0.702; p<0.05). That indicates that the (i.v) admixture training influences the understanding of (i.v) admixture as training tends to be able to obtain information on (i.v) admixtures. That is in line with previous theories that information is also a factor affecting knowledge. The ease with which a person obtain information receives can accelerate a person to acquire new knowledge [7,19].

A statistically significant association was also found between age and knowledge of compounding personnel about (i.v) admixtures (p<0.05). That is related to previous studies whereby an increase in age will increase knowledge [16,20]. With increasing age, the ability to understand and think a person will develop so that the knowledge gained is getting better [7,15]. With the knowledge possessed by personnel, it is hoped that compounding personnel will understand the benefits of (i.v) admixture properly. Our study possesses several strengths including use of a validated survey instrument and the collection of data related to important socio-demographic factors. However, this study has some limitations. This study is quantitative, so some responses may be biased, especially those who complete the survey in front of the researcher. Furthermore, the results of this study cannot be generalized accurately to the entire population. This is because in this study only respondents who fit the inclusion criteria were used, not all health professionals. Therefore, our results are limited to the reported findings.

## Conclusion

Most of the compounding personnel in government hospitals are nurses and have adequate to good knowledge. The age of the respondent and the (i.v) admixture training history are factors that influence the knowledge of the compounding personnel. The knowledge possessed by the compounding personnel is expected to be the basis for the compounding personnel to carry out the procedure of (i.v) admixture appropriately for patient safety.

### What is known about this topic

- According to United Stated Pharmacopeia (USP), the compounding personnel must be trained and have knowledge of (i.v) admixtures;
- Poorly knowledge of the compounding personnel can be a risk to the patient safety.

### What this study adds

- The age of the respondent and the (i.v) admixture training history are factors that influence the knowledge of the compounding personnel about iv admixtures;
- Compounding personnel have poor knowledge on aspects of building and equipment, procedures of (i.v) admixtures and labeling;
- The knowledge of compounding personnel can be increased through the provision of continous training in all aspects regulated by the USP.

## Annex

**Annex 1**: research questionnaire (PDF-377Kb)
